# Polymorphisms of protamine genes contribute to male infertility susceptibility in the Chinese Han population

**DOI:** 10.18632/oncotarget.18660

**Published:** 2017-06-27

**Authors:** Weijun Jiang, Peiran Zhu, Jing Zhang, Qiuyue Wu, Weiwei Li, Shuaimei Liu, Mengxia Ni, Maomao Yu, Jin Cao, Yi Li, Yingxia Cui, Xinyi Xia

**Affiliations:** ^1^ Department of Reproduction and Genetics, Institute of Laboratory Medicine, Jinling Hospital, Nanjing University School of Medicine, Nanjing 210002, P.R. China

**Keywords:** polymorphism, protamine, male infertility, transition protein

## Abstract

Protamine (PRM) plays important roles in the packaging of DNA within the sperm nucleus. To investigate the role of *PRM1/2* and transition protein 1 (*TNP1*) polymorphisms in male infertility, 636 infertile men and 442 healthy individuals were recruited into this case-controlled study of the Chinese Han population, using MassARRAY technology to analyze genotypes. Our analysis showed that there were no significant differences between controls and infertile cases among the five single nucleotide polymorphisms identified in *PRM1*, *PRM2* and *TNP1* [rs737008 (G/A), rs2301365 (C/A), rs2070923 (C/A), rs1646022 (C/G) and rs62180545 (A/G)]. However, we found that the *PRM1* and *PRM2* haplotypes GCTGC, TCGCA and TCGCC exhibited significant protective effects against male infertility compared to fertile men, while TCGGA, GCTCC and TCGGC represented significant risk factors for spermatogenesis. Our data showed that rs737008 and rs2301365 in *PRM1,* and rs1646022 in *PRM2,* were significantly associated with male infertility and that gene–gene interaction played a role in male infertility. A linkage disequilibrium plot for the five SNPs showed that rs737008 was strongly linked with both rs2301365 and rs2070923. These findings are likely to help improve our understanding of the etiology of male infertility. Further studies should include a larger number of genes and SNPs, particularly growing critical genes; such studies will help us to unravel the effect of individual genetic factors upon male infertility.

## INTRODUCTION

Globally, approximately 15% of heterosexual couples suffer from infertility [1–6], in which non-obstructive azoospermia (NOA) and severe oligozoospermia represent two of the predominant phenotypes relating to severely defective spermatogenesis. Although several factors can lead to male infertility, including malformations of the reproductive tract (e.g., varicocele or cryptorchidism, hypogonadotrophic hypogonadism, karyotype anomalies and Y chromosome microdeletions), infection, and chemical exposure, the effect of genetic predisposition upon male infertility remains to be fully clarified [2]. Therefore, an understanding of the genetic basis of reproductive failure is essential in managing an infertile couple appropriately.

Protamines (PRM), which were first isolated from spermatozoa one century ago, play essential roles in sperm chromatin condensation [7]. Two types of protamine were identified in mammals: protamine 1 (PRM1), which is present in vertebrate species, and protamine 2 (PRM2), which only exists in some mammalian species, including humans and mice [8]. *PRM1* and *PRM2* (NC_000016.9, GI: 224589807) are closely linked in a stretch of DNA 13–15 kb long on human chromosome 16p13.3, along with the gene encoding transition protein 2 (TNP2); collectively, these genes are categorized as members of the protamine gene family [2]. Previous research has shown that the biological and physiological functions of protamine are involved in a variety of mechanisms: (i) paternal genome packing; (ii) competition and removal of transcription factors and other proteins from the spermatid; and (iii) imprinting of the paternal genome during spermatogenesis [2, 7]. Mutations or polymorphisms within *PRM* induce conformational changes of the encoded proteins and alter their incorporation into sperm chromatin, leading to sperm defects, although the underlying mechanisms remain largely unknown [8, 9]. Considered as one of the most perplexing disorders in the reproductive field, male factor infertility is prevalent, and its incidence is rising; worryingly, however, the etiology of this condition remains largely elusive.

Since the association between *PRM* polymorphisms and the risk of male infertility was first reported in 2003, there have been additional investigations of the association between rs201365, rs1646022 rs2070923 and rs737008 and the risk of male infertility among different ethnicities [10–16]. However, the results arising from these studies have been mixed or contradictory, most probably due to relatively small sample size. Interestingly, published association studies of male infertility in Chinese populations did not identify SNPs as susceptible loci, perhaps owing to the stringent *P* values required to avoid false-positive findings, which dramatically reduces the possibility of revealing a modest effect of some common SNPs upon male infertility, particularly for those SNPs which are potentially functional. Therefore, in the present study, we further investigated the association of some potentially functional PRM SNPs (rs2301365, rs737008, rs35576928, rs1646022 and rs2070923) and TNP1 (rs62189545) with the risk of male infertility in a large scale study of a Chinese population.

## RESULTS

### Clinical data of the study population

The clinical characteristics of the study population are described in Table 1. Consistently, no significant differences were observed in terms of the relative distributions of age, abstinence time, semen volume, pH of semen and serum hormone index (Testosterone: T, Estradiol: E_2_ and Luteinizing hormone: LH) when considering cases and controls from study sets (*P* > 0.05 for all), with the exception of sperm concentration, progressive mobility and Follicle stimulating estrogen (FSH) status (*P* < 0.05).

**Table 1:** Characteristics of the study population

Characteristics	Case (mean ± SD)	Control (mean ± SD)	*P*
All subjects	636	442	
Age (year)	28.56 ± 4.30	28.37 ± 4.23	0.545
Abstinence time (day)	4.33 ± 1.60	5.08 ± 4.09	0.521
Semen volume (mL)	3.65 ± 1.76	3.51 ± 1.08	0.295
Sperm concentration (10^6^/mL)	12.32 ± 15.49	72.77 ± 45.21	**0.000**
Progressive mobility (%)	15.29 ± 15.06	42.02 ± 9.04	**0.000**
pH value of semen	7.38 ± 0.06	7.37 ± 0.07	0.404
**Serum Hormone Index**			
T (nmol/L)	13.85 ± 5.32	12.43 ± 4.83	0.188
E_2_ (pmol/L)	115.47 ± 67.21	103.87 ± 77.35	0.506
LH (IU/L)	6.28 ± 4.90	4.62 ± 7.49	0.163
FSH (FSH IU/L)	14.69 ± 15.70	4.72 ± 2.51	**0.001**

T: testosterone; E2: estradiol; LH: lutenizing hormone; FSH: follicle-stimulating hormone. Bold font means significant difference when compared with controls (*P* < 0.05)

### Genetic analyses

#### Logistic regression analysis

In the control group, all genotype frequencies of SNPs were in line with the Hardy-Weinberg equilibrium (*P* > 0.05). Genotype distributions of the selected SNPs in cases and controls are summarized in Table 2. For *PRM1*, rs35576928 showed a GG genotype in all samples. Among the five SNPs (rs737008, rs2301365, rs2070923, rs1646022 and rs62180545) for *PRM1*, *PRM2* and *TNP1*, no significant differences were found between the cases and controls. Similarly, no significant differences were observed in the distributions of genotype among cases and controls for all SNPs within subgroups (i.e., NOA, severe oligozoospermia, and other types of infertility).

**Table 2:** Logistic regression analysis of associations between the genotype of *PRM1/2* and *TNP1* and male infertility risk

Genotype	Control	Case			NOA and SO			Other infertility		
		N	*P*	OR(95% CI)	N	*P*	OR(95% CI)	N	*P*	OR(95% CI)
**PRM1 rs737008 230G>T**										
GG	237	339	0.332	ref	298	0.463	ref	41	0.216	ref
GT	159	250	0.567	1.10(0.80-1.53)	205	0.877	1.03(0.73-1.45)	45	0.133	1.63(0.86-3.08)
TT	46	47	0.233	0.71(0.40-1.25)	41	0.246	0.71(0.39-1.27)	6	0.627	0.73(0.21-2.59)
GT/TT	205	297	0.937	1.01(0.74-1.38)	246	0.780	0.96(0.69-1.32)	51	0.257	1.43(0.77-2.64)
**PRM1 rs2301365 c.-190C>A**										
CC	277	378	0.655	ref	329	0.790	ref	49	0.448	ref
CA	144	229	0.357	1.17(0.84-1.62)	192	0.508	1.12(0.80-1.58)	37	0.245	1.46(0.77-2.78)
AA	21	29	0.884	1.06(0.50-2.22)	23	0.942	0.97(0.44-2.13)	6	0.463	1.63(0.44-6.03)
CA/AA	165	258	0.520	1.11(0.81-1.52)	215	0.556	1.10(0.79-1.53)	43	0.209	1.48(0.80-2.75)
**PRM2 rs2070923 373T>G**										
TT	233	333	0.210	ref	292	0.307	ref	41	0.241	ref
TG	162	258	0.510	1.12(0.81-1.55)	213	0.774	1.05(0.75-1.48)	45	0.162	1.57(0.83-2.97)
GG	47	45	0.153	0.66(0.38-1.17)	39	0.162	0.66(0.36-1.19)	6	0.577	0.70(0.20-2.47)
TG/GG	209	303	0.937	1.01(0.74-1.38)	252	0.810	0.96(0.70-1.33)	51	0.310	1.38(0.74-2.54)
**PRM2 rs1646022 298C>G**										
CC	235	335	0.157	ref	284	0.208	ref	51	0.538	ref
CG	166	266	0.466	1.13(0.82-1.56)	229	0.432	1.14(0.82-1.60)	37	0.913	1.04(0.55-1.96)
GG	41	35	0.115	0.61(0.33-1.13)	31	0.170	0.64(0.33-1.21)	4	0.285	0.44(0.10-1.97)
CG/GG	207	301	0.875	1.03(0.75-1.40)	260	0.793	1.04(0.76-1.44)	41	0.789	0.92(0.50-1.70)
**TNP1 rs62180545 c.139+75A>G**										
AA	389	577	0.511	ref	489	0.734	ref	88	0.337	ref
AG	52	57	0.247	0.74(0.44-1.23)	53	0.436	0.81(0.48-1.37)	4	0.141	0.34(0.28-1.44)
GG	1	2	0.983	0.97(0.60-15.57)	2	0.924	1.14(0.07-18.39)	0	1.000	-
AG/GG	53	59	0.253	0.75(0.45-1.23)	55	0.453	0.82(0.49-1.37)	4	0.131	0.33(0.08-1.40)

ref: reference; CI: confidence interval, OR: odds ratio. The results were in bold, if the 95% CI excluded 1 or *P* < 0.05; NOA: non-obstructive azoospermia; SO: severe oligozoospermia.

#### Haplotype analysis

**Figure 1:**
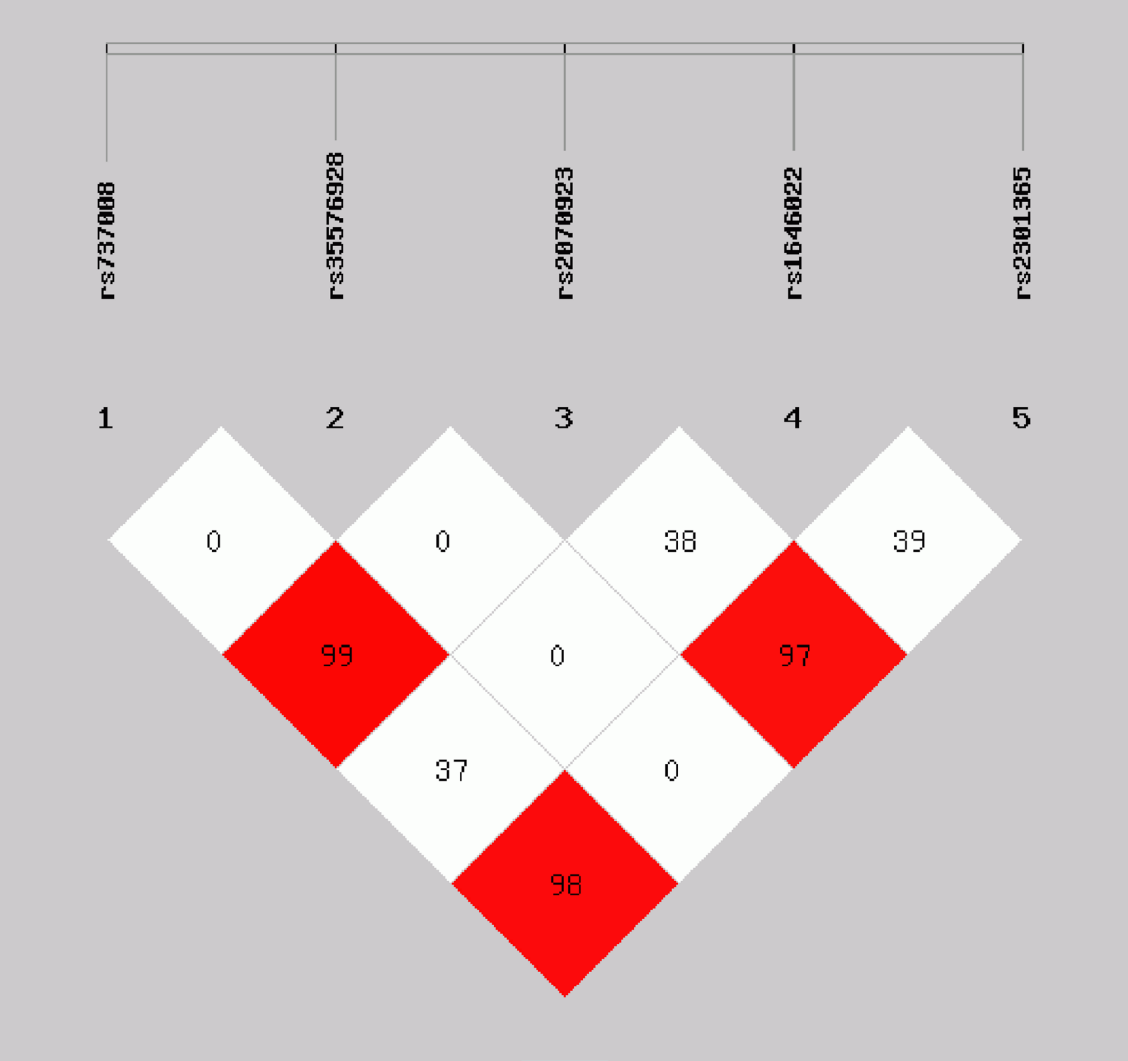
Linkage disequilibrium (LD) plot for five SNPs of *PRM1* and *PRM2* The D value is displayed as a percentage. One LD block has been identified and shows the involvement of five SNPs. Data showed that rs737008 was strongly linked with both rs2301365 and rs2070923.

One of the goals of the present study was to establish the most shared haplotypes present in the *PRM1* and *PRM2* genes in the Chinese Han population, taking advantage of the proximity of the two protamine genes and the fact that we have detected three common SNPs in *PRM1* and two common SNPs in *PRM2*. With this information, we defined five haplotypes present in 97.3% of the chromosomes in the Han population, and six additional rare haplotypes present in 2.7% of the chromosomes in our population (Table 3). A linkage disequilibrium (LD) plot for the five SNPs is shown in Figure 1, indicating that rs737008 was strongly linked with both rs2301365 and rs2070923.

**Table 3:** Haplotype analysis of five SNPs of *PRM1/2*

Haplotype	Control	Case			NOA and SO			Other infertility		
		N	*P*	OR(95% CI)	N	*P*	OR(95% CI)	N	*P*	OR(95% CI)
**rs737008/rs35576928/rs2070923/rs1646022/rs2301365**										
GCTCC	0.492	0.696	**0.000**	**2.40 (1.91-3.02)**	0.712	**0.000**	**2.61(2.05-3.33)**	0.581	0.131	1.41(0.90-2.19)
GCTGC	0.216	0.026	**0.000**	**0.10 (0.06-0.16)**	0.015	**0.000**	**0.05(0.03-0.11)**	0.111	**0.016**	**0.45(0.23-0.87)**
TCGCA	0.167	0.026	**0.000**	**0.13(0.08-0.22)**	0.006	**0.000**	**0.03(0.01-0.09)**	0.148	0.618	0.86(0.47-1.57)
TCGCC	0.057	0.008	**0.000**	**0.13(0.05-0.33)**	0.006	**0.000**	**0.11(0.04-0.32)**	0.026	0.211	0.44(0.12-1.64)
TCGGA	0.041	0.196	**0.000**	**5.73(3.71-8.84)**	0.207	**0.000**	**6.18(3.98-9.58)**	0.118	**0.002**	**3.09(1.48-6.48)**
TCGGC	0.02	0.04	**0.036**	**2.03(1.04-3.96)**	0.042	**0.026**	**2.14(1.08-4.23)**	0.016	-	-

The statistically significant results were in bold, if the 95% CI excluded 1 or *P* < 0.05.

The frequency of the shared haplotypes was significantly different between infertile patients and healthy individuals (*P* < 0.05). As shown in Table 3, six haplotypes were found to be associated with male infertility and especially for the subgroups of NOA and severe oligozoospermia. Three haplotypes exhibited significantly protective effects against male infertility when compared with controls (for the GCTGC haplotype: *P* = 0.000, OR = 0.10, 95%CI = 0.06–0.16; for the TCGCA haplotype: *P* = 0.000, OR = 0.13, 95%CI = 0.08–0.22; for the TCGCC haplotype: *P* = 0.000, OR = 0.10, 95%CI = 0.05–0.33). However, three haplotypes were associated with an increased risk of male infertility (for the TCGGA haplotype: *P* = 0.000, OR = 5.73, 95%CI = 3.71–8.84; for the GCTCC haplotype: *P* = 0.000, OR = 2.40, 95%CI = 1.91–3.02; for the TCGGC haplotype: *P* = 0.036, OR = 2.03, 95%CI = 1.04–3.96). In the subgroup analysis of NOA and severe oligozoospermia, a similar positive effect was found. Nevertheless, in the subgroup analysis of other types of infertility, we unfortunately failed to find a similar effect across several haplotypes, except for the GCTGC haplotype (*P* = 0.016, OR = 0.45, 95%CI = 0.23–0.87) and the TCGGA haplotype (*P* = 0.002, OR = 3.09, 95%CI = 1.48–6.48).

#### Gene–gene interaction analysis

We further evaluated the associations between the combined types of the selected shared SNPs and male infertility (Table 4 and Supplementary Table 1). Individuals with a combined genotype of CGGT (rs1646022/rs737008) were associated with male infertility susceptibility, accompanied by a 1.58-fold increased risk of infertility (*P* = 0.047, OR = 1.58, 95%CI = 1.01–2.47). At the same time, individuals with the CGAC genotype (rs1646022/rs2301365) were associated with male infertility, accompanied by a 1.68-fold increased risk of infertility (*P* = 0.023, OR = 1.68, 95%CI = 1.07–2.64).

**Table 4:** Gene–gene interactions of *PRM1* and *PRM2* and male infertility risk

Genotype	Control	Case			NOA and SO			Other infertility		
		N	OR(95%CI)	*P*	N	OR(95%CI)	*P*	N	OR(95%CI)	*P*
**rs1646022/rs737008**										
GGCC	115	308	ref	0.000	280	ref	0.000	28	ref	0.559
CCGT	94	23	**0.09(0.05-0.18)**	**0.000**	4	**0.02(0.00-0.07)**	**0.000**	19	0.87(0.36-2.08)	0.753
CCTT	27	4	**0.05(0.01-0.23)**	**0.000**	0	-	0.998	4	0.60(0.13-2.86)	0.521
CGGG	96	29	**0.11(0.06-0.21)**	**0.000**	16	**0.07(0.03-0.14)**	**0.000**	13	0.59(0.23-1.55)	0.284
CGGT	53	225	**1.58(1.01-2.47)**	**0.047**	201	1.55(0.98-2.45)	0.059	24	1.85(0.78-4.36)	0.162
CGTT	16	12	**0.27(0.10-0.74)**	**0.011**	12	**0.30(0.11-0.81)**	0.180	0	-	0.999
GGGG	26	2	**0.03(0.00-0.21)**	**0.001**	2	**0.03(0.00-0.24)**	**0.001**	0	-	0.998
GGGT	12	2	**0.06(0.01-0.48)**	**0.008**	0	-	0.989	2	0.67(0.08-5.68)	0.711
GGTT	3	31	4.28(0.96-19.06)	0.056	29	4.41(0.98-19.74)	0.053	2	3.00(0.26-35.33)	0.383
**rs1646022/rs2301365**										
CCCC	139	309	ref	0.000	278	ref	0	31	ref	0.756
CCAA	15	4	**0.13(0.03-0.60)**	**0.009**	0	-	0.999	4	1.28(0.26-6.36)	0.767
CCAC	83	22	**0.12(0.06-0.23)**	**0.000**	6	**0.04(0.01-0.12)**	**0.000**	16	0.84(0.34-2.07)	0.699
CGAA	5	6	0.48(0.11-2.21)	0.349	6	0.54(0.12-2.46)	0.425	0	-	0.999
CGAC	52	193	**1.68(1.07-2.64)**	**0.023**	174	**1.68(1.07-2.66)**	**0.026**	19	1.68(0.70-4.02)	0.246
CGCC	108	67	**0.27(0.17-0.44)**	**0.000**	49	**0.22(0.13-038)**	**0.000**	18	0.72(0.30-1.710	0.453
GGAA	1	19	6.46(0.81-51.19)	0.078	17	6.47(0.81-51.83)	0.079	2	6.38(0.38-107.11)	0.198
GGAC	9	14	0.65(0.22-1.90)	0.426	12	0.62(0.20-1.89)	0.396	2	0.91(0.11-7.90)	0.932
GGCC	30	2	**0.03(0.00-0.22)**	**0.001**	2	**0.03(0.00-0.25)**	**0.001**	0	-	0.998

The statistically significant results were in bold, if the 95% CI excluded 1 or *P* < 0.05.

For rs737008 in *PRM1* and rs1646022 in *PRM2*, we found a protective effect for male infertility in several combined genotypes (for GTCC: *P* = 0.000, OR = 0.09, 95%CI = 0.05–0.18; for TTCC: *P* = 0.000, OR = 0.05, 95%CI = 0.01–0.23; for GGCG: *P* = 0.000, OR = 0.11, 95%CI = 0.06–0.21; for GTCG: *P* = 0.011, OR = 0.27, 95%CI = 0.10–0.74; for GGGG: *P* = 0.001, OR = 0.03, 95%CI = 0.00–0.21; for GTCG: *P* = 0.008, OR = 0.06, 95%CI = 0.01–0.48). Similarly, for rs2301365 in *PRM1* and rs1646022 in *PRM2*, there was a significant protective effect for male infertility in several combined genotypes (for AACC: *P* = 0.009, OR = 0.13, 95%CI = 0.03–0.60; for ACCC: *P* = 0.000, OR = 0.12, 95%CI = 0.06–0.23; for CCCG: *P* = 0.000, OR = 0.27, 95%CI = 0.17–0.44; for CCGG: *P* = 0.001, OR = 0.03, 95%CI = 0.00–0.22). However, we failed to find a significant difference for other combined genotypes (Table 4 and Supplementary Table 1). The basic information of six SNPs was described in the Supplementary Table 2.

## DISCUSSION

In mammals, male germ cells differentiate from haploid round spermatids into flagella-possessing motile sperm in a process called spermiogenesis [17, 18]. This process is different from somatic cell differentiation in that the majority of the core histones are replaced sequentially, first by transition proteins and then by protamines, thus facilitating chromatin hyper-compaction [7, 18, 19]. This histone-to-protamine transition process represents an excellent model for the investigation of how epigenetic regulators interact with each other to remodel chromatin architecture [20–22].

Thus far, only a few studies have analyzed the correlation between *PRM1/2* and *TNP1* polymorphisms and particular phenotypes of male infertility [10, 13, 15, 16, 23, 24]. However, recent studies in the field have highlighted the critical role of rs2301365 (c.-190 C>A), located in the 5′-UTR of the *PRM1* gene, in controlling haploid-specific developmental programming [2, 9, 11, 15]. Similarly, Jiang et al.,[2] reported that rs737008 (c.230 C>A) and rs1646022 (c.298 G>C) had strong protective effects over male infertility in several subgroups. However, no significant difference was found for other SNPs when comparing infertile cases and controls. Other studies investigated the role of several SNPs in male infertility, but failed to detect any differences in allele frequency between infertile patients and healthy men [2, 13, 14, 23, 25, 26].

These previous results may be attributable to a multitude of factors. Firstly, studies with relatively small sample sizes may lack the necessary power to allow accurate conclusions to be drawn. Secondly, the SNPs investigated may be located on non-sensitive sites. We cannot exclude the possibility that these SNPs may simply represent genetic markers of male infertility in linkage disequilibrium with other mutations or variations, which do play a role in male infertility. Thirdly, the settings and experimental methods differ across different studies. Finally, races living in different latitudes with extreme weather are under the influence of the environment, climatic conditions (air temperature, solar radiation, ultraviolet intensity) and varied dietary habits during the lengthy evolution process, which may affect the mode of action and potency of the SNPs, thus leading to differences in results among populations from certain regions [22, 27–29].

Haplotype analysis is a useful tool in revealing the potential associations of genes that are hidden by the evaluation of *PRM1* and *PRM2* SNPs individually. The present study identified high linkage disequilibrium among the selected SNPs. We identified five haplotypes that were present in 97.3% of the chromosomes in the Han population, and six additional rare haplotypes that were present in 2.7% of the chromosomes in our population. Several shared SNPs were strongly associated with rs737008 rs2301365 and rs2070923. Three haplotypes (GCTGC, TCGCA and TCGCC) were shown to be significant protective genetic factors for spermatogenesis, compared with the controls. In addition, three haplotypes (TCGGA, GCTCC and TCGGC) were associated with an increased risk of spermatogenesis and male infertility. In the subgroup analysis of NOA and severe oligozoospermia, similar positive effects were also found. Nevertheless, in the subgroup analysis of other types of infertility, we unfortunately failed to find a similar effect for several haplotypes, except for GCTGC and TCGGA. The potential mechanisms underlying these observations need to be investigated further.

Using gene–gene interaction analysis, we successfully examined 12 combined genotypes that were significantly associated with male infertility. The combined genotypes of CGGT (rs1646022/rs737008) and CGAC (rs1646022/rs2301365) were two risk factors for male infertility, accompanied by a 1.58–1.68-fold increased risk of infertility. We also found an intensely beneficial effect upon male infertility for several combined genotypes of rs737008 and rs1646022, such as GTCC, TTCC, GGCG, GTCG and GGGG. Similarly, a significant protective role for male infertility was identified for several combined genotypes of rs737008 and rs1646022 was found, such as AACC, ACCC, CCCG, and CCGG. Previous studies have shown that rs737008 and rs2301365 in *PRM1,* and rs1646022 in *PRM2,* were significantly associated with male infertility. This is because interference in the expression of PRM1/2 is essential for normal spermatogenesis. These results suggested that several SNPs in *PRM1/2* might be an independent risk factor for male infertility.

Our study has several strengths and limitations. Based on a case-controlled study, we have obtained a rewarding result in that we identified an association between several SNPs and male infertility. However, there are also some limitations. Firstly, selection bias is unavoidable on account of the hospital-based case-controlled nature of this study. Secondly, with restriction to a China Han population, it is uncertain whether our findings could be generalized to other populations. We should therefore aim to analyze samples from several regions and ethnicities in future [29, 30]. Lastly, due to technological limitations, we could not verify the function of the selected SNPs, which may have helped us to understand the precise molecular mechanisms underlying the function of these selected SNPs and their influencing upon male infertility [31, 32].

In conclusion, we found that the *PRM1* and *PRM2* haplotypes GCTGC, TCGCA and TCGCC exhibited significant protective effects against male infertility as compared to fertile men, except for TCGGA, GCTCC and TCGGC which could represent a significant increased risk of spermatogenesis. Our study showed that rs737008 and rs2301365 in *PRM1,* and rs1646022 in *PRM2,* were significantly associated with male infertility and that gene–gene interactions played a role in male infertility. These findings will help us to further understand the aetiology of male infertility. Further studies should include a larger number of patients, genes, and SNPs, particularly growing critical genes; this will ultimately help us to unravel the effects of individual genetic factors upon the development of male infertility.

## MATERIALS AND METHODS

### Ethics statement

All patients provided written informed consent for the collection of samples and their subsequent analysis. This study was conducted in accordance with the tenets of the Declaration of Helsinki and its amendments, and was approved by the ethics committee of Jinling Hospital, Nanjing University.

### Study population

This case-controlled study recruited 693 infertile men for a genetic questionnaire. All patients attended the Reproductive Medicine Centre of Jinling Hospital of Nanjing University between July 2013 and January 2015. At least two semen samples were analyzed for each patient to confirm the diagnosis of NOA or severe oligozoospermia. NOA was defined as a zero sperm count after semen centrifugation at 3000g for 10 min while severe oligozoospermia was defined as the detection limit of sperm concentration of < 2 × 10^6^/ml and when the proportion of progressive sperm was < 32%.

All patient diagnoses were based upon a comprehensive andrological examination including medical history, physical examination, semen analysis, hormone analysis, karyotype analysis and Y chromosome microdeletion screening. Patients with a history of varicocele, epididymitis, orchitis, hypogonadotrophic hypogonadism, mono- or bilateral cryptorchidism, obstruction/absence of the vas deferens, chromosomal abnormalities, and Y chromosome microdeletions, were carefully excluded. Among the 693 infertile men, ten cases of chromosomal abnormalities, and 47 cases of Y chromosome microdeletions, were excluded from the final genotype analysis. Thus, 636 study cases were finally investigated (544 cases with NOA and severe oligozoospermia and 92 cases with other types of infertility).

The control group consisted of 442 hospital-based normozoospermia men aged from 24 to 45 years, who were recruited from the same reproductive medicine center, where they were in search of assisted reproduction technology because of female infertility. All controls had fathered at least one child previously. From each patient, we collected a range of clinical characteristics, including age, semen quantity, sperm counts, hormone levels, and PR value.

### Genetic analyses

A panel of six SNPs of *PRM1*, *PRM2* and *TNP1* were selected for study, based on previous investigations. Genotyping analysis of the SNPs selected for fast-track validation analysis was performed using Sequenom MassARRAY technology (San Diego, CA, USA). Genomic DNA (15 ng) was used to genotype each sample. Locus-specific PCR and detection primers were designed using MassARRAY Assay Design 3.0 software at the Department of Reproduction and Genetics at the affiliated Jinling Hospital of Nanjing University. DNA samples were amplified by multiplex PCRs and the amplification products were then used for locus-specific single-base extension reactions. The resulting products were desalted and transferred to a 384-elementSpectroCHIP array (Sequenom). Allele detection was performed using matrix-assisted laser desorption/ionization time-of-flight mass spectrometry (Sequenom). Mass spectrograms were analysed by MassARRAY Typer software. SNPs detected with a call rate lower than 90% in the cases and controls, or beyond the Hardy–Weinberg equilibrium in the controls (*P* < 0.05), were excluded.

### Statistical analysis

Differences in the distributions of demographic characteristics, selected variables, and frequencies of genotypes between cases and controls were tested by the Student’s *t* test (for continuous variables) or chi-square-test (for categorical variables). Hardy–Weinberg equilibrium was determined based upon the control genotyping results. A logistic regression analysis was used to analyze genotype distributions in the cases and controls. Linkage disequilibria and haplotypes were analysed with SHEsis software (http://analysis.bio-x.cn/myAnalysis.php; Shi and He, 2005). Frequencies of < 0.03 were ignored in the linkage disequilibrium analysis. The association between polymorphisms and the risk of infertility was analyzed by calculating the odds ratios (OR) and 95% confidence intervals (CI) and by comparing allele frequency or genotype of the case group with that of the control group. Strict Bonferroni correction was used to correct the *P*-values of the allele frequency and genotype for each SNP. Multiplicative interactions were assessed by logistic regression or Cox regression. The corrected significance level was interpreted as 0.0125 after Bonferroni correction for multiple comparisons. All tests were two-sided and carried out with SPSS software (version 22; SPSS Institute). *P* < 0.05 was considered statistically significant.

## SUPPLEMENTARY MATERIALS TABLES





## Abbreviations

PRM: Protamine
TNP1: Transition protein
SNP: Single nucleotide polymorphisms
NOA: Non-obstructive azoospermia
LD: Linkage disequilibrium plot
OR: odds ratios
95%CI: 95% confidence intervals
T: Testosterone
E_2_: Estradiol
LH: Lutenizing hormone
FSH: Follicle-stimulating hormone
SO: Severe oligozoospermia
