# Supplementary material for: Polymorphisms of protamine genes contribute to male infertility susceptibility in the Chinese Han population
Source: Oncotarget. 2017 Jun 27;8(37):61637–45. doi: 10.18632/oncotarget.18660 (PMC5617452; doi:10.18632/oncotarget.18660)
Supplement: Supplementary file 1 [file oncotarget-08-61637-s001.pdf]

Polymorphisms of protamine genes contribute to male infertility susceptibility in the Chinese Han population

SUPPLEMENTARY MATERIALS

Supplementary Table 1: Gene-gene interactions of *TNP1* and *PRM1/2* and male infertility risk

See Supplementary File 1

Supplementary Table 2: Relevant information about the five single nucleotide polymorphisms of *PRM1/2* and *TNP1* gene

SNP	Chromosome	Location	Nucleotide change	Amino acid change	P_{HWE}	MAF
**rs737008**	16p13.3	Exon	G>T	Arg47Arg	0.061	0.270
**rs2301365**	16p13.3	5'-UTR	C>A		0.682	0.226
**rs35576928**	16p13.3	Exon	G>T	Ser34Arg	1.00	0.000
**rs2070923**	16p13.1	Intron	T >G		0.061	0.201
**rs1646022**	16p13.1	Exon	C>G	Ala100Pro	0.143	0.195
**rs62180545**	2q14.2	Intron	A>G		0.590	0.042
