# Supplementary material for: Polymorphisms of protamine genes contribute to male infertility susceptibility in the Chinese Han population
Source: Oncotarget. 2017 Jun 27;8(37):61637–45. doi: 10.18632/oncotarget.18660 (PMC5617452; doi:10.18632/oncotarget.18660)
Supplement: Supplementary file 2 [file oncotarget-08-61637-s002.docx]

**Supplementary Table 1** **Gene-gene interactions of** ***TNP1* and *PRM1/2*** **and** **male infertility risk**

| **Genotype** | **Control** | **Case** | | | **NOA and SO** | | | **Other infertility** | | |
| --- | --- | --- | --- | --- | --- | --- | --- | --- | --- | --- |
|  |  | N | *P* | OR(95% CI) | N | *P* | OR(95% CI) | N | *P* | OR(95% CI) |
| **rs62180545/rs35576928** |  |  |  |  |  |  |  |  |  |  |
| AACC | 389 | 577 | 0.511 | ref | 489 | 0.734 | ref | 88 | 0.216 | ref |
| AGCC | 51 | 56 | 0.983 | 1.03(0.06-16.57) | 54 | 0.924 | 0.87(0.05-14.05) | 4 | 1.000 | - |
| GGCC | 2 | 3 | 0.851 | 0.76(0.05-12.72) | 3 | 0.812 | 0.71(0.04-11.87) | 0 | 1.000 | - |
| **rs62180545/rs737008** |  |  |  |  |  |  |  |  |  |  |
| AAGG | 209 | 314 | 0.677 | ref | 275 | 0.834 | ref | 39 | 0.566 | ref |
| AAGT | 139 | 221 | 0.717 | 1.07(0.75-1.51) | 179 | 0.919 | 0.98(0.68-1.41) | 42 | 0.129 | 1.66(0.86-3.20) |
| AATT | 41 | 43 | 0.250 | 0.71(0.39-1.28) | 38 | 0.252 | 0.70(0.38-1.29) | 5 | 0.688 | 0.77(0.22-2.76) |
| AGGG | 27 | 25 | 0.209 | 0.63(0.30-1.30) | 23 | 0.278 | 0.66(0.31-1.40) | 2 | 0.364 | 0.39(0.05-3.03) |
| AGGT | 19 | 27 | 0.923 | 0.96(0.44-2.09) | 25 | 0.958 | 1.02(0.46-2.25) | 2 | 0.573 | 0.55(0.07-4.41) |
| AGTT | 5 | 4 | 0.402 | 0.48(0.09-2.67) | 4 | 0.494 | 0.55(0.99-3.05) | 0 | 0.999 | - |
| GGGT | 2 | 2 | 0.979 | 0.26(0.06-15.5) | 2 | 0.946 | 1.10(0.07-17.75) | 0 | 1.000 | - |
| **rs62180545/rs2301365** |  |  |  |  |  |  |  |  |  |  |
| AACC | 243 | 349 | 0.923 | ref | 301 | 0.979 | ref | 48 | 0.738 | ref |
| AAAA | 19 | 27 | 0.989 | 1.01(0.47-2.17) | 23 | 0.828 | 0.91(0.40-2.07) | 4 | 0.486 | 1.59(0.43-5.97) |
| AAAC | 126 | 202 | 0.545 | 1.11(0.79-1.58) | 166 | 0.745 | 1.06(0.74-1.53) | 36 | 0.276 | 1.44(0.74-2.79) |
| AGAA | 2 | 2 | 0.997 | 1.01(0.06-12.2) | 2 | 0.916 | 1.16(0.07-18.74) | 0 | 1.000 | - |
| AGAC | 18 | 25 | 0.989 | 1.01(0.45-2.23) | 23 | 0.863 | 1.07(0.48-2.42) | 2 | 0.600 | 0.57(0.07-4.58) |
| AGCC | 32 | 29 | 0.179 | 0.63(0.32-1.24) | 27 | 0.272 | 0.68(0.34-1.36) | 2 | 0.263 | 0.31(0.04-2.40) |
| GGAC | 0 | 2 | 1.000 | - | 2 | 1.000 | - | 0 | 1.000 | - |
| GGCC | 2 | 0 | 1.000 | - | 0 | 1.000 | - | 0 | 1.000 | - |
| **rs62180545/rs2070923** |  |  |  |  |  |  |  |  |  |  |
| AATT | 206 | 307 | 0.589 | ref | 268 | 0.775 | ref | 39 | 0.580 | - |
| AAGG | 42 | 42 | 0.166 | 0.66(0.36-1.19) | 26 | 0.168 | 0.64(0.35-1.20) | 6 | 0.636 | 0.74(0.21-2.63) |
| AAGT | 140 | 229 | 0.590 | 1.10(0.79-1.56) | 186 | 0.901 | 1.02(0.71-1.47) | 43 | 0.147 | 1.62(0.84-3.13) |
| AGGG | 5 | 4 | 0.460 | 0.48(0.09-2.68) | 4 | 0.500 | 0.56(0.10-3.08) | 0 | 0.999 | - |
| AGGT | 21 | 27 | 0.794 | 0.90(0.42-1.94) | 25 | 0.921 | 0.96(0.44-2.09) | 2 | 0.521 | 0.51(0.06-4.04) |
| AGTT | 26 | 25 | 0.275 | 0.66(0.32-1.39) | 23 | 0.358 | 0.70(0.33-1.50) | 2 | 0.384 | 0.40(0.05-3.15) |
| GGGT | 2 | 2 | 0.982 | 0.97(0.06-15.62) | 2 | 0.942 | 1.11(0.07-17.91) | 0 | 1.000 | - |
| **rs62180545/rs1646022** |  |  |  |  |  |  |  |  |  |  |
| AACC | 208 | 310 | 0.651 | ref | 260 | 0.803 | ref | 50 | 0.856 | ref |
| AACG | 146 | 237 | 0.640 | 1.09(0.77-1.53) | 201 | 0.610 | 1.10(0.77-1.57) | 36 | 0.953 | 1.02(0.53-1.96) |
| AAGG | 34 | 31 | 0.159 | 0.62(0.32-1.21) | 27 | 0.214 | 0.64(0.32-1.29) | 4 | 0.351 | 0.49(0.11-2.20) |
| AGCC | 26 | 25 | 0.275 | 0.66(0.32-1.39） | 23 | 0.410 | 0.73(0.34-1.55) | 2 | 0.280 | 0.32(0.04-2.51) |
| AGCG | 19 | 27 | 0.935 | 0.97(0.45-2.10) | 25 | 0.870 | 1.07(0.49-2.35) | 2 | 0.434 | 0.44(0.06-3.47) |
| AGGG | 7 | 4 | 0.261 | 0.39(0.07-2.23) | 4 | 0.358 | 0.46(0.09-2.41) | 0 | 0.999 | - |
| GGCC | 2 | 0 | 1.000 | - | 0 | 1.000 | - | 0 | 1.000 | - |
| GGCG | 0 | 2 | 1.000 | - | 2 | 1.000 | - | 0 | 0.000 | - |
